# 3,4-Dinitro-2,5-bis­[4-(trifluoro­meth­yl)phen­yl]thio­phene

**DOI:** 10.1107/S1600536809020443

**Published:** 2009-06-06

**Authors:** Ping-Hsin Huang, Jiun-Yi Shen, Yuh-Sheng Wen

**Affiliations:** a Cardinal Tien College of Healthcare and Management, Taipei 231, Taiwan; b Institute of Chemistry, Academia Sinica, Nankang, Taipei, Taiwan

## Abstract

The title compound, C_18_H_8_F_6_N_2_O_4_S, is a precursor for the production of low-band-gap conjugated polymers. In the crystal structure, the dihedral angles between the thio­phene and benzene rings are 35.90 (8) and 61.94 (8)°, and that between the two benzene rings is 40.18 (8)°. The two nitro groups are twisted with respect to the thio­phene ring, the dihedral angles being 53.66 (10) and 31.63 (10)°. Weak inter­molecular C—H⋯O hydrogen bonding helps to stabilize the crystal structure.

## Related literature

For a related structure, see: Bak *et al.* (1961 ▶).
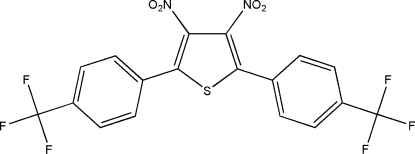


## Experimental

### 

#### Crystal data

C_18_H_8_F_6_N_2_O_4_S
*M* *_r_* = 462.32
Orthorhombic, 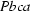

*a* = 8.1572 (3) Å
*b* = 17.6371 (6) Å
*c* = 24.4150 (8) Å
*V* = 3512.6 (2) Å^3^
*Z* = 8
Mo *K*α radiation
μ = 0.28 mm^−1^
*T* = 100 K
0.4 × 0.36 × 0.1 mm

#### Data collection

Bruker SMART CCD area-detector diffractometer
Absorption correction: multi-scan (*SADABS*; Bruker, 2001 ▶) *T*_min_ = 0.895, *T*_max_ = 0.973
22650 measured reflections
3098 independent reflections
1888 reflections with *I* > 2σ(*I*)
*R*_int_ = 0.058

#### Refinement

*R*[*F* ^2^ > 2σ(*F* ^2^)] = 0.030
*wR*(*F* ^2^) = 0.053
*S* = 0.80
3098 reflections
281 parameters
H-atom parameters constrained
Δρ_max_ = 0.29 e Å^−3^
Δρ_min_ = −0.28 e Å^−3^

### 

Data collection: *SMART* (Bruker, 2001 ▶); cell refinement: *SAINT* (Bruker, 2001 ▶); data reduction: *SAINT*; program(s) used to solve structure: *SHELXS97* (Sheldrick, 2008 ▶); program(s) used to refine structure: *SHELXL97* (Sheldrick, 2008 ▶); molecular graphics: *ORTEP-3 for Windows* (Farrugia, 1997 ▶); software used to prepare material for publication: *WinGX* (Farrugia, 1999 ▶).

## Supplementary Material

Crystal structure: contains datablocks global, I. DOI: 10.1107/S1600536809020443/xu2528sup1.cif

Structure factors: contains datablocks I. DOI: 10.1107/S1600536809020443/xu2528Isup2.hkl

Additional supplementary materials:  crystallographic information; 3D view; checkCIF report

## Figures and Tables

**Table 1:** Hydrogen-bond geometry (Å, °)

*D*—H⋯*A*	*D*—H	H⋯*A*	*D*⋯*A*	*D*—H⋯*A*
C13—H13⋯O4^i^	0.93	2.52	3.320 (2)	144

Symmetry code: (i) 
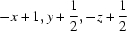
.

**Fig. 1.**
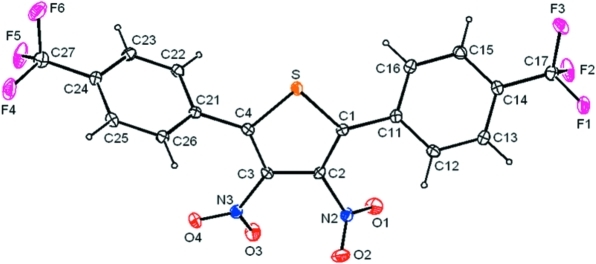
A molecular structure of (I) with 30% probability displacement ellipsoids, showing the atom-numbering scheme employed. H atoms are shown as small spheres of the arbitrary radii.

**Fig. 2.**
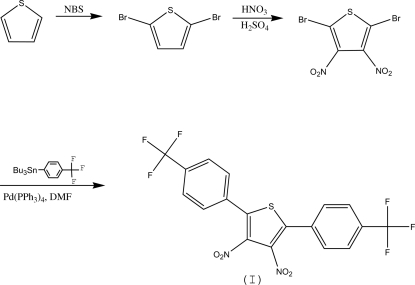
The formation of the title compound.

## supplementary crystallographic information

### Comment

The title compound, (I), has been shown to be an excellent precursor for the production of low band gap conjugated polymers and organic light-emitting devices *etc*. As indicated in Scheme 2, standard procedures were administrated to synthesize in high yield. The molecular structure is shown in Fig. 1. The double bonds and C—C single bond of (I) are slightly shorter than those of the parent thiophene, while the S—C single bond is slightly elongated (Bak *et al.*, 1961). The dihedral angles between the thiophene (S/C1–C4) and benzene rings (C11–C16 and C21–C26) are 35.90 (8) and 61.94 (8)°, respectively, and that between the two benzene rings is 40.18 (8)°. The two nitro groups are oriented at the thiophene ring with the dihedral angles of 53.66 (10) and 31.63 (10)°, respectively. Intermolecular weak C—H···O hydrogen bonding helps to stabilize the crystal structure (Table 1).

### Experimental

The compound was synthesized by the following procedure. A two-necked round-bottomed flask was charged with Pd(PPh_3_)_4_ (280 mg), tributyl(4-(trifluoromethyl)phenyl)stannane (3.26 g, 7.5 mmol), 2,5-dibromo-3,4-dinitrothiophene (1.00 g, 3.0 mmol) and DMF (20 ml), and the reaction mixture stirred under nitrogen and heated at 343 K for 48 h. After cooling, the mixture was diluted with diethyl ether and the organic phase was washed with water and brine. After drying over anhydrous MgSO_4_ and removing the volatiles, the residue was purified by column chromatography using CH_2_Cl_2_/n-hexane as eluent, followed by recrystallization from CH_2_Cl_2_ and hexane to yield 0.7 g (50%) of (I) as a white solid. Crystals suitable for X-ray diffraction were grown from a CH_2_Cl_2_ solution layered with hexane at room temperature.

### Refinement

H atoms were located geometrically and treated as riding atoms, with C—H = 0.93 Å, and with *U*_iso_(H) = 1.2*U*_eq_(C).

### Crystal data

**Table:**

C_18_H_8_F_6_N_2_O_4_S	*F*(000) = 1856
*M**_r_* = 462.32	*D*_x_ = 1.748 Mg m^−^^3^
Orthorhombic, *P**b**c**a*	Mo *K*α radiation, λ = 0.71073 Å
Hall symbol: -P 2ac 2ab	Cell parameters from 3689 reflections
*a* = 8.1572 (3) Å	θ = 2.3–21.2°
*b* = 17.6371 (6) Å	µ = 0.28 mm^−^^1^
*c* = 24.4150 (8) Å	*T* = 100 K
*V* = 3512.6 (2) Å^3^	Plate, colourless
*Z* = 8	0.4 × 0.36 × 0.1 mm

### Data collection

**Table:**

Bruker SMART CCD area-detector diffractometer	3098 independent reflections
Radiation source: fine-focus sealed tube	1888 reflections with *I* > 2σ(*I*)
graphite	*R*_int_ = 0.058
ω and φ scans	θ_max_ = 25.0°, θ_min_ = 1.7°
Absorption correction: multi-scan (*SADABS*; Bruker, 2001)	*h* = −8→9
*T*_min_ = 0.895, *T*_max_ = 0.973	*k* = −20→20
22650 measured reflections	*l* = −29→29

### Refinement

**Table:**

Refinement on *F*^2^	Secondary atom site location: difference Fourier map
Least-squares matrix: full	Hydrogen site location: inferred from neighbouring sites
*R*[*F*^2^ > 2σ(*F*^2^)] = 0.030	H-atom parameters constrained
*wR*(*F*^2^) = 0.053	*w* = 1/[σ^2^(*F*_o_^2^) + (0.0222*P*)^2^] where *P* = (*F*_o_^2^ + 2*F*_c_^2^)/3
*S* = 0.80	(Δ/σ)_max_ < 0.001
3098 reflections	Δρ_max_ = 0.28 e Å^−^^3^
281 parameters	Δρ_min_ = −0.28 e Å^−^^3^
0 restraints	Extinction correction: *SHELXL97* (Sheldrick, 2008), Fc^*^=kFc[1+0.001xFc^2^λ^3^/sin(2θ)]^-1/4^
Primary atom site location: structure-invariant direct methods	Extinction coefficient: 0.00042 (7)

### Special details

**Table:**

Experimental. ^1^H NMR (CDCl_3_): 7.77 (d, J = 8.2, 4H), 7.64 (d, J = 8.2, 4H). FAB MS (m/e): 462 (*M*^+^). Analysis calculated for C_18_H_8_F_6_N_2_O_4_S: C 46.76, H 1.74, N 6.06%; found: C 46.80, H 1.88, N 5.79%.
Geometry. All e.s.d.'s (except the e.s.d. in the dihedral angle between two l.s. planes) are estimated using the full covariance matrix. The cell e.s.d.'s are taken into account individually in the estimation of e.s.d.'s in distances, angles and torsion angles; correlations between e.s.d.'s in cell parameters are only used when they are defined by crystal symmetry. An approximate (isotropic) treatment of cell e.s.d.'s is used for estimating e.s.d.'s involving l.s. planes.
Refinement. Refinement of *F*^2^ against ALL reflections. The weighted *R*-factor *wR* and goodness of fit *S* are based on *F*^2^, conventional *R*-factors *R* are based on *F*, with *F* set to zero for negative *F*^2^. The threshold expression of *F*^2^ > σ(*F*^2^) is used only for calculating *R*-factors(gt) *etc*. and is not relevant to the choice of reflections for refinement. *R*-factors based on *F*^2^ are statistically about twice as large as those based on *F*, and *R*- factors based on ALL data will be even larger.

### Fractional atomic coordinates and isotropic or equivalent isotropic displacement parameters (Å^2^)

**Table:**

	*x*	*y*	*z*	*U*_iso_*/*U*_eq_	
S	0.49028 (6)	0.16607 (3)	0.10370 (2)	0.01968 (14)	
F1	0.35605 (13)	0.58240 (6)	0.14011 (4)	0.0308 (3)	
F2	0.55441 (13)	0.58514 (6)	0.08266 (5)	0.0355 (3)	
F3	0.31448 (13)	0.55311 (6)	0.05612 (4)	0.0317 (3)	
F4	0.39559 (15)	−0.25593 (7)	0.10705 (5)	0.0436 (4)	
F5	0.59498 (14)	−0.24370 (7)	0.05129 (5)	0.0414 (4)	
F6	0.35364 (14)	−0.21365 (7)	0.02679 (5)	0.0421 (4)	
O1	0.84762 (17)	0.29479 (8)	0.21049 (6)	0.0336 (4)	
O2	0.72134 (16)	0.23172 (8)	0.27348 (6)	0.0327 (4)	
O3	0.89423 (17)	0.10570 (8)	0.23361 (6)	0.0326 (4)	
O4	0.72044 (15)	0.01316 (8)	0.23007 (5)	0.0227 (4)	
N2	0.75230 (19)	0.24606 (10)	0.22593 (7)	0.0206 (4)	
N3	0.7648 (2)	0.07741 (10)	0.21850 (6)	0.0198 (4)	
C1	0.5770 (2)	0.23578 (11)	0.14335 (7)	0.0154 (5)	
C2	0.6654 (2)	0.20278 (11)	0.18389 (8)	0.0152 (5)	
C3	0.6626 (2)	0.12295 (11)	0.18302 (7)	0.0151 (5)	
C4	0.5731 (2)	0.09335 (10)	0.14100 (7)	0.0145 (5)	
C11	0.5395 (2)	0.31580 (10)	0.13231 (8)	0.0153 (5)	
C12	0.5170 (2)	0.36726 (11)	0.17431 (8)	0.0177 (5)	
H12	0.5278	0.3514	0.2105	0.021*	
C13	0.4788 (2)	0.44149 (11)	0.16326 (8)	0.0182 (5)	
H13	0.4654	0.4758	0.1918	0.022*	
C14	0.4601 (2)	0.46509 (11)	0.10993 (8)	0.0169 (5)	
C15	0.4800 (2)	0.41410 (11)	0.06754 (8)	0.0213 (5)	
H15	0.4659	0.4299	0.0315	0.026*	
C16	0.5204 (2)	0.34036 (11)	0.07853 (8)	0.0203 (5)	
H16	0.5353	0.3064	0.0498	0.024*	
C17	0.4211 (2)	0.54560 (12)	0.09785 (9)	0.0225 (5)	
C21	0.5441 (2)	0.01459 (11)	0.12329 (8)	0.0154 (5)	
C22	0.5951 (2)	−0.00879 (11)	0.07186 (8)	0.0191 (5)	
H22	0.6485	0.0252	0.0488	0.023*	
C23	0.5672 (2)	−0.08169 (11)	0.05477 (8)	0.0204 (5)	
H23	0.6030	−0.0973	0.0204	0.024*	
C24	0.4859 (2)	−0.13201 (11)	0.08861 (7)	0.0167 (5)	
C25	0.4314 (2)	−0.10885 (11)	0.13930 (8)	0.0182 (5)	
H25	0.3749	−0.1424	0.1618	0.022*	
C26	0.4607 (2)	−0.03585 (11)	0.15652 (8)	0.0171 (5)	
H26	0.4242	−0.0203	0.1908	0.021*	
C27	0.4586 (3)	−0.21090 (12)	0.06936 (9)	0.0249 (5)	

### Atomic displacement parameters (Å^2^)

**Table:**

	*U*^11^	*U*^22^	*U*^33^	*U*^12^	*U*^13^	*U*^23^
S	0.0205 (3)	0.0148 (3)	0.0238 (3)	−0.0007 (3)	−0.0048 (3)	0.0006 (2)
F1	0.0375 (8)	0.0209 (7)	0.0339 (8)	0.0100 (6)	0.0032 (6)	−0.0016 (6)
F2	0.0225 (7)	0.0184 (7)	0.0654 (9)	−0.0028 (6)	0.0090 (6)	0.0108 (6)
F3	0.0315 (7)	0.0291 (8)	0.0345 (8)	0.0085 (6)	−0.0084 (6)	0.0057 (6)
F4	0.0709 (10)	0.0208 (7)	0.0391 (8)	−0.0164 (7)	0.0180 (7)	−0.0038 (6)
F5	0.0265 (8)	0.0240 (7)	0.0738 (10)	0.0025 (6)	0.0122 (7)	−0.0171 (7)
F6	0.0447 (8)	0.0355 (8)	0.0462 (9)	−0.0044 (6)	−0.0134 (7)	−0.0163 (7)
O1	0.0313 (9)	0.0344 (10)	0.0351 (9)	−0.0202 (8)	−0.0052 (8)	0.0047 (8)
O2	0.0415 (10)	0.0389 (10)	0.0176 (9)	−0.0115 (8)	−0.0001 (8)	−0.0018 (8)
O3	0.0219 (9)	0.0289 (9)	0.0469 (10)	−0.0039 (7)	−0.0169 (8)	−0.0003 (7)
O4	0.0227 (9)	0.0182 (8)	0.0271 (9)	−0.0001 (7)	0.0004 (7)	0.0057 (7)
N2	0.0183 (11)	0.0192 (11)	0.0244 (12)	0.0025 (9)	−0.0038 (9)	−0.0018 (9)
N3	0.0189 (11)	0.0199 (11)	0.0206 (10)	0.0016 (9)	0.0001 (9)	−0.0022 (9)
C1	0.0117 (11)	0.0178 (12)	0.0167 (11)	−0.0027 (9)	0.0006 (9)	−0.0006 (10)
C2	0.0112 (11)	0.0169 (12)	0.0177 (12)	−0.0046 (10)	0.0003 (9)	−0.0032 (10)
C3	0.0109 (12)	0.0179 (12)	0.0166 (12)	0.0014 (10)	0.0006 (10)	0.0029 (10)
C4	0.0093 (11)	0.0180 (12)	0.0163 (11)	0.0018 (9)	0.0024 (9)	0.0021 (10)
C11	0.0083 (11)	0.0163 (12)	0.0213 (12)	−0.0042 (9)	0.0005 (10)	0.0001 (10)
C12	0.0155 (12)	0.0197 (12)	0.0179 (11)	−0.0020 (10)	−0.0018 (10)	0.0029 (10)
C13	0.0146 (12)	0.0175 (12)	0.0226 (12)	−0.0021 (10)	0.0025 (10)	−0.0028 (10)
C14	0.0101 (11)	0.0158 (12)	0.0248 (12)	−0.0031 (9)	−0.0007 (10)	0.0020 (10)
C15	0.0216 (12)	0.0206 (13)	0.0217 (12)	−0.0025 (11)	−0.0021 (10)	0.0030 (10)
C16	0.0214 (12)	0.0175 (12)	0.0220 (12)	−0.0015 (11)	0.0013 (10)	−0.0032 (10)
C17	0.0189 (13)	0.0217 (13)	0.0268 (14)	0.0010 (11)	0.0009 (11)	−0.0012 (11)
C21	0.0106 (12)	0.0166 (12)	0.0190 (12)	0.0024 (9)	−0.0041 (9)	0.0000 (9)
C22	0.0172 (12)	0.0196 (13)	0.0205 (12)	−0.0027 (10)	0.0001 (10)	0.0036 (10)
C23	0.0193 (12)	0.0240 (13)	0.0179 (12)	−0.0014 (10)	0.0025 (10)	−0.0039 (10)
C24	0.0109 (11)	0.0160 (11)	0.0233 (12)	0.0002 (10)	−0.0014 (10)	−0.0021 (9)
C25	0.0131 (12)	0.0182 (12)	0.0232 (12)	0.0006 (9)	0.0016 (10)	0.0045 (10)
C26	0.0151 (12)	0.0181 (12)	0.0182 (11)	0.0039 (10)	0.0015 (10)	−0.0002 (9)
C27	0.0233 (14)	0.0249 (13)	0.0266 (13)	−0.0003 (11)	0.0048 (12)	−0.0020 (11)

### Geometric parameters (Å, °)

**Table:**

S—C4	1.7119 (19)	C12—C13	1.373 (2)
S—C1	1.7173 (19)	C12—H12	0.9300
F1—C17	1.329 (2)	C13—C14	1.375 (2)
F2—C17	1.344 (2)	C13—H13	0.9300
F3—C17	1.346 (2)	C14—C15	1.381 (2)
F4—C27	1.320 (2)	C14—C17	1.485 (3)
F5—C27	1.330 (2)	C15—C16	1.368 (3)
F6—C27	1.347 (2)	C15—H15	0.9300
O1—N2	1.2187 (19)	C16—H16	0.9300
O2—N2	1.2147 (18)	C21—C26	1.383 (2)
O3—N3	1.2249 (18)	C21—C22	1.386 (2)
O4—N3	1.2226 (18)	C22—C23	1.371 (3)
N2—C2	1.462 (2)	C22—H22	0.9300
N3—C3	1.446 (2)	C23—C24	1.382 (3)
C1—C2	1.356 (2)	C23—H23	0.9300
C1—C11	1.469 (2)	C24—C25	1.377 (2)
C2—C3	1.408 (2)	C24—C27	1.485 (3)
C3—C4	1.363 (2)	C25—C26	1.375 (2)
C4—C21	1.474 (2)	C25—H25	0.9300
C11—C12	1.382 (2)	C26—H26	0.9300
C11—C16	1.391 (2)		
			
C4—S—C1	94.24 (9)	C15—C16—C11	120.54 (18)
O2—N2—O1	125.10 (17)	C15—C16—H16	119.7
O2—N2—C2	117.48 (17)	C11—C16—H16	119.7
O1—N2—C2	117.40 (17)	F1—C17—F2	106.49 (16)
O4—N3—O3	124.26 (17)	F1—C17—F3	106.33 (16)
O4—N3—C3	118.87 (17)	F2—C17—F3	105.24 (16)
O3—N3—C3	116.82 (17)	F1—C17—C14	113.47 (17)
C2—C1—C11	131.07 (18)	F2—C17—C14	112.20 (16)
C2—C1—S	108.86 (14)	F3—C17—C14	112.51 (17)
C11—C1—S	119.92 (14)	C26—C21—C22	119.21 (18)
C1—C2—C3	114.15 (17)	C26—C21—C4	120.86 (17)
C1—C2—N2	123.10 (18)	C22—C21—C4	119.88 (17)
C3—C2—N2	122.72 (18)	C23—C22—C21	120.34 (19)
C4—C3—C2	113.78 (17)	C23—C22—H22	119.8
C4—C3—N3	123.16 (18)	C21—C22—H22	119.8
C2—C3—N3	122.46 (18)	C22—C23—C24	120.00 (18)
C3—C4—C21	131.90 (18)	C22—C23—H23	120.0
C3—C4—S	108.95 (14)	C24—C23—H23	120.0
C21—C4—S	119.11 (14)	C25—C24—C23	120.11 (18)
C12—C11—C16	118.74 (18)	C25—C24—C27	120.92 (18)
C12—C11—C1	121.51 (17)	C23—C24—C27	118.97 (18)
C16—C11—C1	119.71 (17)	C26—C25—C24	119.76 (18)
C13—C12—C11	120.72 (17)	C26—C25—H25	120.1
C13—C12—H12	119.6	C24—C25—H25	120.1
C11—C12—H12	119.6	C25—C26—C21	120.55 (18)
C12—C13—C14	119.99 (18)	C25—C26—H26	119.7
C12—C13—H13	120.0	C21—C26—H26	119.7
C14—C13—H13	120.0	F4—C27—F5	107.18 (18)
C13—C14—C15	119.97 (19)	F4—C27—F6	105.61 (16)
C13—C14—C17	120.08 (18)	F5—C27—F6	105.08 (16)
C15—C14—C17	119.94 (18)	F4—C27—C24	113.67 (17)
C16—C15—C14	120.02 (18)	F5—C27—C24	112.76 (17)
C16—C15—H15	120.0	F6—C27—C24	111.91 (18)
C14—C15—H15	120.0		

### Hydrogen-bond geometry (Å, °)

**Table:**

*D*—H···*A*	*D*—H	H···*A*	*D*···*A*	*D*—H···*A*
C13—H13···O4^i^	0.93	2.52	3.320 (2)	144

Symmetry codes: (i) −*x*+1, *y*+1/2, −*z*+1/2.
